# What are karrikins and how were they ‘discovered’ by plants?

**DOI:** 10.1186/s12915-015-0219-0

**Published:** 2015-12-21

**Authors:** Gavin R. Flematti, Kingsley W Dixon, Steven M. Smith

**Affiliations:** School of Chemistry and Biochemistry, University of Western Australia, Perth, Western Australia 6009 Australia; Department of Environment and Agriculture, Curtin University, Bentley, Perth, Western Australia 6102 Australia; School of Plant Biology, University of Western Australia, Perth, Western Australia 6009 Australia; School of Biological Sciences, University of Tasmania, Hobart, Tasmania 7001 Australia; Institute of Genetics and Developmental Biology, Chinese Academy of Sciences, Beijing, 100101 China

## Abstract

Karrikins are a family of compounds produced by wildfires that can stimulate the germination of dormant seeds of plants from numerous families. Seed plants could have ‘discovered’ karrikins during fire-prone times in the Cretaceous period when flowering plants were evolving rapidly. Recent research suggests that karrikins mimic an unidentified endogenous compound that has roles in seed germination and early plant development. The endogenous signalling compound is presumably not only similar to karrikins, but also to the related strigolactone hormones.

## What are karrikins?

**Fig. 1.**
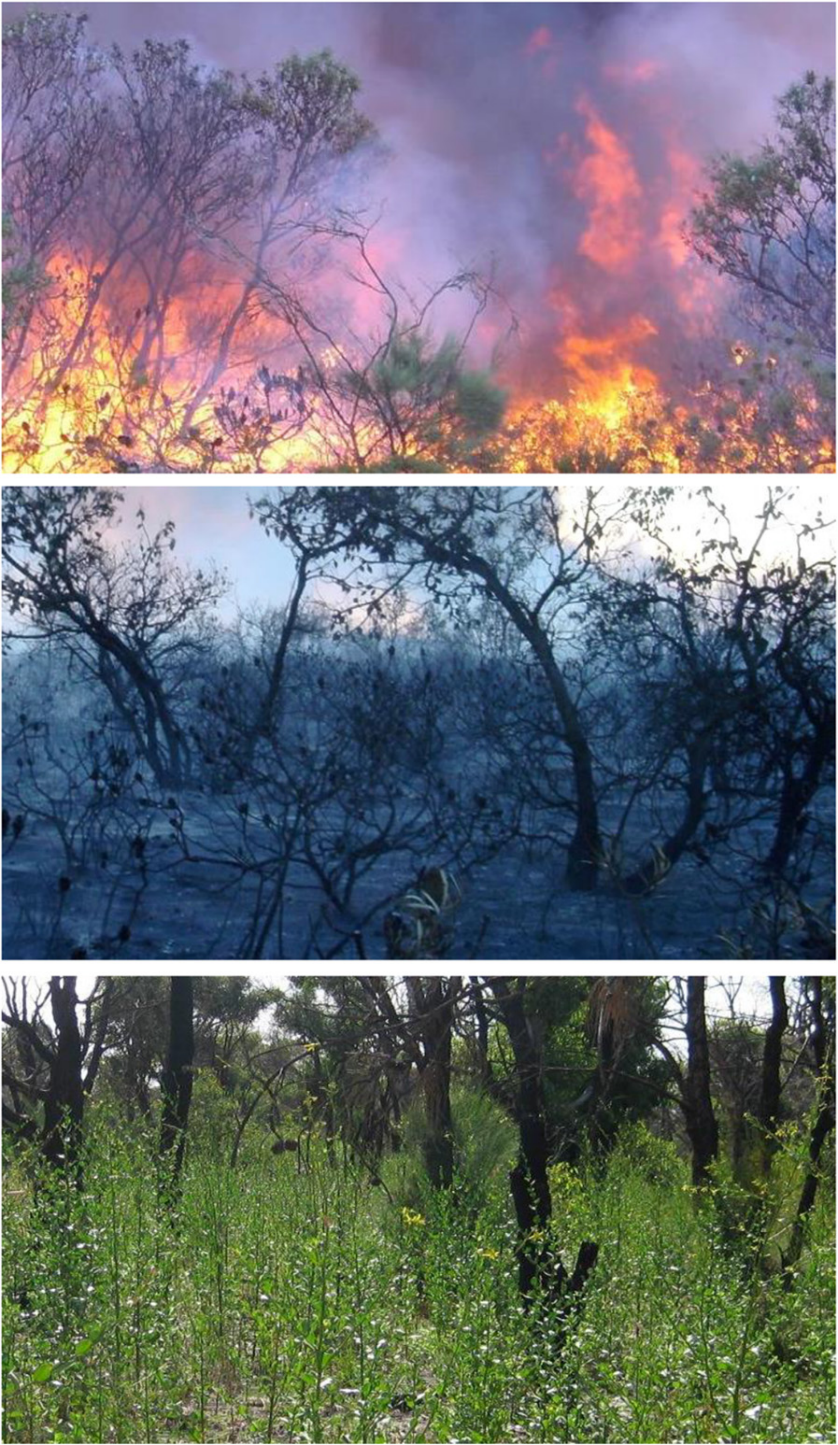
The role of karrikins in revegetation after a fire. A bushfire generates smoke and ash containing karrikins (*upper panel*). After the fire karrikins are present on the soil surface (*middle panel*). After the first rains, the karrikins stimulate germination of the soil seed bank and the growth of new plants, in this case *Anthocercis littorea* (*lower panel*). Top and middle photographs with permission from Vanessa Westcott (Bush Heritage Australia) and bottom photograph from the authors

Karrikins are a family of closely related small organic compounds that are produced when plant material burns [1]. They are remarkable because they can stimulate the seeds of many plant species to germinate *en masse*. In particular, some plants that grow immediately after bushfires or wildfires have evolved such that their seeds remain dormant in the soil until a fire generates karrikins that are bound to soil particles. Then, once the karrikins are washed into the soil by rainfall, they stimulate the seeds to germinate. Some of these species will grow rapidly, flower, produce new seed and then die, usually after a year or two (Fig. 1). The new seed that falls to the ground remains dormant until a future fire generates fresh karrikins to awaken them to grow and produce a new generation of seeds. Such plants are referred to as ‘fire-followers’ or ‘fire-ephemerals’ — because they are ‘temporary’. This is a very specialised but successful strategy, because fires release plant-bound nutrients and create an open habitat where seedlings can thrive and become established before other competing plants colonise the landscape. One remarkable feature of this trait is that rain without a previous fire does not stimulate seeds of fire-ephemerals to germinate. Therefore, such seeds can undergo many cycles of wetting (imbibition) and drying (dehydration) but remain dormant and karrikin-responsive. Secondly, it means that such seeds can remain viable in the soil for decades between fires.

## How were karrikins discovered?

**Fig. 2.**
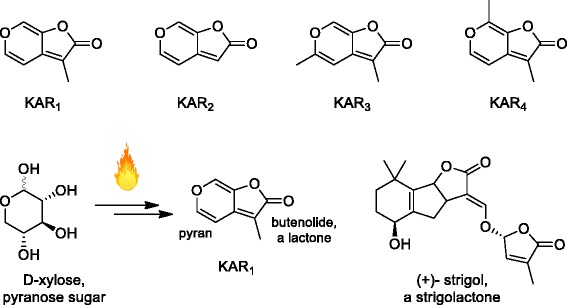
The karrikin family. The first karrikin discovered was KAR_1_, also known as karrikinolide. Since karrikins can be produced by burning sugars such as xylose, the pyran ring of karrikins is probably derived from such pyranose sugars. Both karrikins and strigolactone hormones such as strigol have a butenolide ring

It was known for many years that seeds of some fire-followers were stimulated to germinate not by the heat of a fire but by the chemicals it produced [1]. Smoke could achieve the same effect as fire, as could water in which char was soaked or through which smoke was passed, producing ‘smoke water’ [2]. A number of groups around the world searched for the active compound, which involved separating the thousands of compounds in smoke water into different fractions by liquid chromatography and testing each fraction for seed-germination activity. Active fractions were further fractionated and assayed. This approach of bioassay-guided fractionation eventually led to the isolation of an active compound, which was confirmed through chemical synthesis [3]. This compound contains a specific type of lactone known as a butenolide fused to a pyran ring with the systematic name 3-methyl-2*H*-furo[2,3-*c*]pyran-2-one. Subsequently, several closely related compounds were discovered in smoke and collectively referred to as ‘karrikins’ (Fig. 2).

## Where does the karrikin name come from?

The formal names of the compounds are too complex for common usage and there are many butenolides in nature, so a more recognisable common name was required. To reflect their discovery in smoke, the family of compounds was named ‘karrikins’ from the word ‘karrik’, which is an Aboriginal term for smoke from the Western Australian Noongar people [4, 5]. It is common practice in biology to add the ‘-in’ suffix to denote a group of related molecules, such as ‘cytokinins’ in plants or ‘endorphins’ in animals. Fire and smoke play important roles in Aboriginal culture in Australia, and the karrikin name acknowledges that fact. The original compound identified is often referred to as ‘karrikinolide’: the ‘-olide’ suffix indicates that it is a lactone. The karrikins are abbreviated to KAR and numbered in order of their identification in smoke (Fig. 2).

## What are the properties of karrikins?

Karrikins comprise only C, H and O, and contain two ring structures, one of which is a pyran and the other a lactone comprising a five-membered ring known as a butenolide. The pure compound is a crystalline substance with a melting point of 118–119 °C that readily dissolves in organic solvents and sparingly in water. All the karrikins are closely related in structure. The first of the compounds to be identified, KAR_1_, is usually the most abundant in smoke and the most active in seed germination. Seeds of some fire-followers can respond to as little as 10^−10^ M KAR_1_, which is similar in effectiveness to plant hormones [1].

## How are karrikins produced by fire?

It was found that burning many different plant materials, including straw, cellulose filter paper and even sugars, could generate karrikins. Their production from polysaccharides and sugars explains the pyran ring of karrikins, which is proposed to be derived directly from pyranose sugars in plant material (Fig. 2). The precise chemical reaction is unknown but it requires oxygen, and it is even possible to generate seed-germination activity by heating plant material at 180 °C for 30 minutes. So karrikins are probably produced around the home by toasting and roasting certain plant foods. The smoke from cigarettes stimulates seed germination, probably due to the presence of karrikins. Further research has shown that karrikins are unstable at very high temperatures [6]. It is likely, therefore, that they are produced in the less-intense parts of wildfires, vaporise, and collect in the smoke and condensation and become bound to soil particles in the same way that cooled smoke can be deposited onto seeds to stimulate their germination. Karrikins may be ‘carried’ in smoke by a process of steam distillation but are not carried for long distances in smoke, and largely remain close to the source of the fire [1].

## How long do karrikins remain in the soil?

Measurements of karrikins in soil are technically very challenging but seed-germination bioassays can be used to detect activity, one study suggesting that active compound(s) can persist in the soil for over seven years after a fire [7]. Karrikins are unstable in ultraviolet light [6] so they might be expected to decay rapidly in natural sunlight; however, smoke contains many aromatic compounds that can absorb ultraviolet light and could protect karrikins by acting as organic ‘sunscreens’. On the other hand, karrikins can be washed away by rain and elute through sandy soils relatively quickly, so their concentration will steadily decline.

## Why do karrikins not stimulate new seed to germinate?

It might be expected that seeds falling from a new plant in a burnt landscape would immediately encounter karrikins in the soil, and so germinate. While this happens for just a few species, most species need their seeds to be buried in soil for a year or more, a process known as after-ripening, before they become karrikin-responsive. There is also evidence that some seeds may need to undergo a series of wetting and drying cycles in the soil before they become responsive, which means that they depend on a future fire for germination [1].

## What types of plants respond to karrikins?

Seeds from many different families of flowering plants and conifers representing many plant life forms (trees, shrubs, herbs, annuals) will respond to karrikins, and many more respond to smoke, implying that the karrikin response is widespread and may have evolved independently in different groups [1]. Plants with smoke-responsive seed are found in both fire-prone and non-fire-prone environments. Most are dicotyledonous plants but many grasses also respond to smoke. Surprisingly, not only fire-followers respond but also many weedy species, including agricultural weeds [8]. Even seeds of horticultural plants such as lettuce and tomato will have improved germination in response to karrikins under some circumstances. This implies that the ability or potential for a response to karrikins is something fundamental to plants, but the fire-followers have fine-tuned this response to their advantage in post-fire landscapes.

## Do fires produce any other chemicals that stimulate germination?

**Fig. 3.**
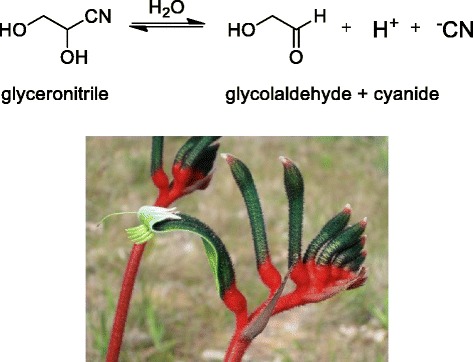
Stimulation of seed germination by cyanohydrins. Cyanohydrins such as glyceronitrile, which was discovered in smoke, are able to stimulate seed germination as a result of hydrolysis to release cyanide. The red and green kangaroo paw *Anigozanthos manglesii* is an example of one fire-follower that responds to cyanohydrins but not to karrikins. Photograph from the authors

Yes, fires do produce other chemicals that stimulate seed germination. It was recognised that a few smoke-responsive species are not karrikin-responsive, one example of which is the stunning red and green kangaroo paw (*Anigozanthos manglesii*), the floral emblem of Western Australia. This led to a search for a new compound that stimulates germination of kangaroo paw, and the discovery of a class of compounds called cyanohydrins [9], an example of which is glyceronitrile (Fig. 3). Cyanohydrins contain nitrogen and slowly hydrolyse to liberate small amounts of cyanide. It is the cyanide that stimulates germination of some seeds. Only a few other fire-following plants do not respond to either karrikins or cyanohydrins [10], so there might be other, minor compounds awaiting discovery, but karrikins are the major germination-stimulating compounds. Other products of fires include nutrients such as nitrate, and this can help to promote seed germination, but it is not fire-specific.

## Do karrikins have any other effects on plants?

**Fig. 4.**
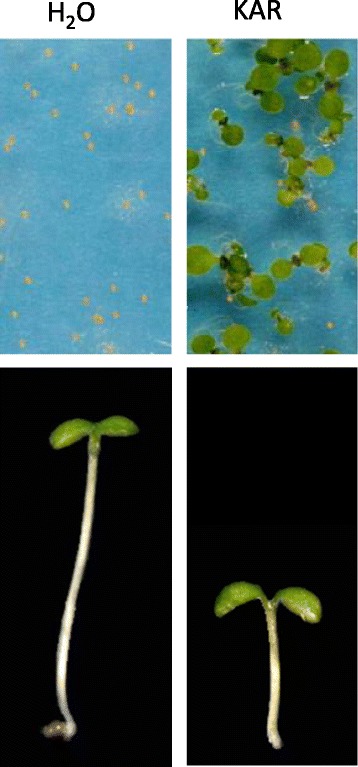
Germination of *Arabidopsis* seeds and growth of seedlings in response to karrikin. *Arabidopsis thaliana* seeds with primary dormancy incubated for seven days on water-agar without karrikin (KAR) germinate very poorly whereas those with KAR germinate readily (top row). Seedlings germinated on nutrient medium and grown in low light for seven days without KAR have long hypocotyls whereas those with KAR have short hypocotyls and larger cotyledons (bottom row). Images from the author’s laboratory

Yes, karrikins will affect seedling growth and development. They are reported to cause more rapid or vigorous growth of some seedlings, including maize and tomato, and in *Arabidopsis thaliana* karrikins influence seedling photomorphogenesis, causing smaller stature seedlings with larger seed leaves [11] (Fig. 4). Such responses in fire followers would enable the seedlings to become rapidly established in the recently burnt environment. Genetic analysis in *Arabidopsis* further suggests that the karrikin response pathway (see below) may control leaf development, but direct effects of karrikins on leaves have not yet been reported.

## Are karrikins of commercial or practical use?

Smoke water is sometimes used to promote germination of garden and horticultural seeds, and can be purchased commercially or easily made. However, many commercial smoke germination products are made from combusting wood that, because of its high lignin content, produces germination inhibitors. Aerosol smoke is also used, and some nurseries or landscape restoration operations use this approach to treat seeds directly or to smoke seedling trays [12]. There has been much interest in the possible use of chemically synthesised karrikins to treat soil to bring about wide-scale and vigorous germination of the resident weed soil seed bank — a process known as ‘suicidal germination’. This could be used for re-vegetation of degraded land, or to promote germination of dormant weed seeds in farmer’s fields so that the weeds can be eliminated. Further research is needed to develop more cost-effective synthesis and improve delivery methods for large-scale commercial application of karrikins.

## How do karrikins work?

The realisation that many plant species respond to karrikins led to the discovery that seeds of *Arabidopsis thaliana* can respond [5]. *Arabidopsis* is the geneticist’s dream because of the resources and knowledge that are available. *Arabidopsis* seeds with a small amount of dormancy will respond to KAR_1_ or KAR_2_, provided that there is no nitrate present, which causes seeds to germinate regardless of the karrikin. Selection of *Arabidopsis* mutants that fail to respond to karrikins led to the discovery of two genes that are essential for karrikin action. One gene, named *MORE AXILARY GROWTH2* (*MAX2*), was already known for its role in responses to strigolactone hormones [13], while the other, *KARRIKIN-INSENSITIVE2* (*KAI2*), was similar to the gene coding for the strigolactone receptor, known as *DWARF14* [11]. These discoveries led to the idea that karrikins simply mimic strigolactones, because they both have a butenolide ring (Fig. 2). We now know that this is not the case in *Arabidopsis*. Karrikins and strigolactones are perceived separately and the plant responds differently to the two classes of compound, but the two systems are obviously very closely related [11]. Formally we still do not know if the mode of action of karrikins in fire-followers is the same as that in *Arabidopsis*, but all plants apparently contain a *KAI2* gene, so it seems likely.

## What is the normal function of the karrikin response system?

**Fig. 5.**
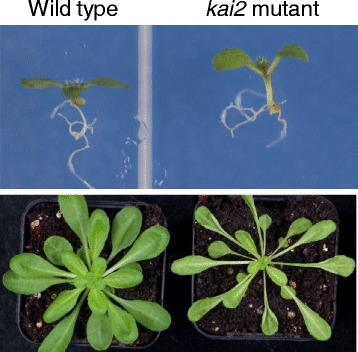
Growth of *Arabidopsis karrikin-insensitive* (*kai2*) mutant. *Arabidopsis thaliana* wild type and *karrikin-insensitive* mutant grown in the light for seven days (*upper panel*) and three weeks (*lower panel*). Images from the author’s laboratory

**Fig. 6.**
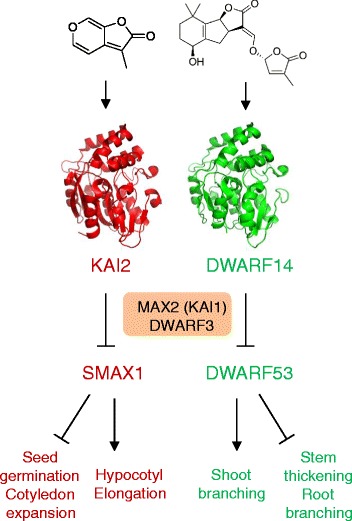
Parallels between karrikin signalling and strigolactone signalling. Karrikins depend on KAI2 protein (shown in *red*) for signalling, while strigolactones signal via interaction with its paralog DWARF14 (*green*). Both are thought to signal by triggering the degradation of closely related proteins, SMAX1 in *Arabidopsis* and DWARF53 in rice, which requires orthologous proteins known as MAX2 (or KAI1) in *Arabidopsis* and DWARF3 in rice. Since DWARF53 and SMAX1 influence different processes, the effects of karrikins and strigolactones on plant development are different. DWARF53 promotes shoot branching, so its degradation in response to strigolactone and DWARF14 inhibit shoot branching. The converse is true for stem thickening. Similar processes operate to regulate seed germination and seedling development by SMAX1

Although many plant species can respond to karrikins when given in high enough doses, they would not all be expected to encounter or to respond to karrikins in nature. *Arabidopsis*, for example, is not a fire-follower but is often observed following physical disturbance of soil (a disturbance species). There could theoretically be other sources of karrikins in the environment that stimulate germination of disturbance species but there is no direct evidence of karrikin production in non-fire soils. The genes for karrikin response are conserved throughout seed plants, which implies that they have a more ancestral or fundamental function. In fact, the *KAI2* gene encoding the proposed karrikin receptor can be traced back to algae and bacteria. Mutant *Arabidopsis* lacking the KAI2 protein have dormant seeds, elongated seedlings and long narrow leaves (Fig. 5). Therefore, this protein has a key function in plant development and presumably responds to an endogenous signalling compound that is similar to karrikins. There is no evidence that plants produce karrikins. The unidentified signalling compound is also likely to be similar to strigolactones, since KAI2 is very similar to the strigolactone receptor DWARF14 [11] (Fig. 6).

## Are karrikins metabolised by plants?

Since evidence for the direct interaction of KAR_1_ with KAI2 is equivocal, there remains the possibility that the karrikin compounds found in smoke require uptake and conversion by the seed into an active compound before interaction with KAI2. This could explain why some seeds are less responsive than others, if they lack the processes and enzymes for uptake or metabolism. Although there are reports that KAR_1_ can interact directly with KAI2 protein, other studies do not support this. A further complication is that while strigolactones are destroyed by the hydrolase activity of their receptor protein DWARF14, such evidence is lacking for karrikins and KAI2. It is possible, therefore, that plants take up karrikins and convert them to active compounds that then interact directly with the KAI2 protein.

## How does the karrikin response system influence plant growth and development?

Treatment of plants with karrikins or analysis of mutants that do not respond to karrikins reveals that signalling though the KAI2 pathway leads to changes in gene expression. A search for mutants that suppress the karrikin-insensitive mutant phenotype led to the discovery of a gene named *SUPPRESSOR OF MAX2-1* (*SMAX1*), which is required for the KAI2 signalling system. At the same time a closely related gene named *DWARF53* was found to be required for strigolactone signalling in rice. These genes encode proteins that are repressors of gene transcription, and DWARF53 is degraded when strigolactones are present, leading to activation of target genes. It is likely that karrikin signalling works in the same way, but targets different genes for activation, with different growth responses (Fig. 6) [14].

## How did plants ‘discover’ karrikins?

**Fig. 7.**
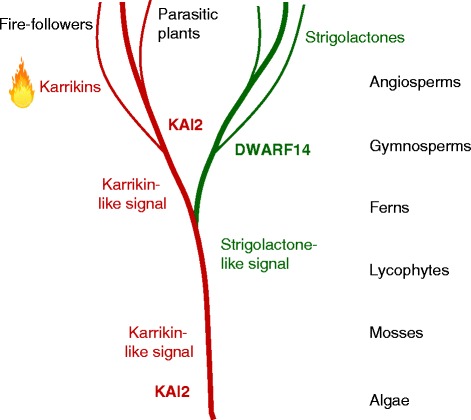
Possible evolution of the karrikin response system in plants. The *KAI2* gene can be traced back to single-celled algae. Gene duplication before the evolution of seed plants leading to *DWARF14* genes probably provided the opportunity for functional specialisation of *KAI2*-type genes, including roles in the response to karrikins and the response of parasitic plants to host-derived strigolactones. The origin of strigolactones is unclear since mosses are reported to produce them. Karrikin-like signals distinct from strigolactones probably have an ancient origin

Fires have been a feature of the Earth since land plants evolved, because they provide fuel and oxygen for fires, and electrical storms or volcanism provide the spark. There is evidence of burnt wood dating back 400 million years ago (Ma). Responses of seed plants to fire probably evolved mainly in the ‘fiery’ Cretaceous period — 65–145 Ma — when conifers dominated and Angiosperms were evolving rapidly [15]. Today, satellite imaging reveals thousands of fires every day, some natural, many caused by humans. So it is not surprising that plants have not only learnt to live with fire, but also to exploit it. Since the *KAI2* gene can be traced back to the earliest plants, its original function was presumably to control aspects of growth and development in response to an endogenous karrikin-like or strigolactone-like signalling compound. This assumption is supported by the observation that a KAI2 protein from the non-seed lycophyte plant *Selaginella* can rescue (or complement) seedling and leaf development in an *Arabidopsis kai2* mutant, but does not respond to either karrikins or strigolactones [16]. It presumably responds to an endogenous signal that has been conserved since before the evolution of seed plants. It is quite easy to imagine, therefore, that some plants evolved so that the KAI2 protein also recognised karrikins from fires, so that they could be used as a specific trigger for post-fire germination. The acquisition of this trait was probably helped by gene duplication events during the evolution of seed plants to create *DWARF14*, so that the *KAI2* gene could be deployed to detect karrikins, while *DWARF14* could be used in other aspects of plant development (Fig. 7). Another example of evolution of these genes for a specific purpose is that of the global parasitic Orobanchaceae family, witchweeds and broomrapes, which are scourges of crops and gardens [13]. In these plants *KAI2*-type genes expressed in the seeds are adapted to detect strigolactones exuded by the roots of host plants, and so trigger germination [17].

## Where can I go for more information?

See references [1–17].
